# Tumor Treating Fields Enhance Temozolomide‐Associated Growth Suppression in Glioblastoma Cells and Patient‐Derived Organoids Through Cell‐Cycle Blockade, Apoptosis, and SMAD‐Associated Signaling

**DOI:** 10.1002/cns.71195

**Published:** 2026-10-09

**Authors:** Haiping Zhu, Yingbo Hou, Xuetao Li, Yi Tang, Zuoyu Jiang, Yang Zhang, Xuewen Zhang, Jiaming Du, Yang Zhu, Hanmiao Dong, Yeyang Xu, Ruoyu Sun, Chenyang Li, Zijian Han, Jian Lu, Lingzhi Xiong, Yulun Huang

**Affiliations:** ^1^ Department of Neurosurgery The Fourth Affiliated Hospital of Soochow University Suzhou China; ^2^ Hunan an Tai Kang Cheng Biotechnology Co. Ltd. Changsha China

**Keywords:** glioblastoma (GBM), patient‐derived organoids (PDOs), SMAD‐associated signaling, temozolomide (TMZ), tumor‐ treating fields (TTF)

## Abstract

**Aims:**

Glioblastoma (GBM) remains difficult to treat despite multimodal therapy. Tumor Treating Fields (TTF) disrupts mitosis and provides clinical benefit in GBM, but the molecular responses associated with TTF and the effects of combining TTF with temozolomide (TMZ) remain incompletely defined.

**Methods:**

TMZ, TTF, and TMZ + TTF were evaluated in U251 and SHG140 GBM cells and patient‐derived organoids. TTF was applied at 200 kHz and 1.75 V/cm RMS. Viability, colony formation, migration‐related phenotypes, apoptosis, cell‐cycle distribution, Western blotting, qRT‐PCR, RNA‐seq, and organoid morphology/immunofluorescence analyses were performed. SB525334 was used to assess SMAD‐associated signaling.

**Results:**

Combined TMZ + TTF treatment more strongly suppressed cell viability, clonogenic growth, and migration‐related phenotypes than either monotherapy, produced greater disruption of organoid structural integrity, and was accompanied by increased cleaved caspase‐3 activation and apoptotic populations. RNA‐seq identified TTF‐associated transcriptomic changes involving chromatin‐associated processes, DNA replication, and cell‐cycle pathways. TTF was associated with selected SMAD‐related signaling changes, G2/M accumulation, altered checkpoint signaling, and enhanced proapoptotic signaling. SB525334 partially attenuated TTF‐associated SMAD phosphorylation, growth suppression, and cleaved caspase‐3 induction.

**Conclusion:**

TTF enhances TMZ‐associated antitumor responses in GBM cell lines and organoids, accompanied by cell‐cycle blockade, apoptosis‐associated changes, and selected SMAD‐related signaling responses.

## Graphical abstract

Tumor Treating Fields enhanced temozolomide‐associated growth suppression in glioblastoma cells and patient‐derived organoids. Combined treatment reduced viability and clonogenic growth, increased apoptosis‐associated responses, and was accompanied by G2/M‐phase accumulation and selected SMAD‐associated signaling changes.

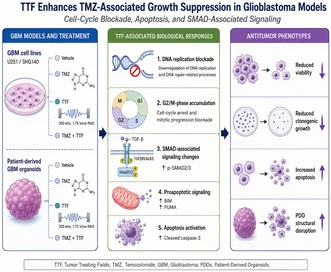

**Abbreviations**
ALK5: activin receptor‐like kinase 5
ANOVA: analysis of variance
ATP: adenosine triphosphate
CCK‐8: Cell Counting Kit‐8
DAPI: 4′,6‐diamidino‐2‐phenylindole
DEGs: differentially expressed genes
DMSO: dimethyl sulfoxide
EMT: epithelial–mesenchymal transition
FBS: fetal bovine serum
FDR: false discovery rate
GBM: glioblastoma
GO: Gene Ontology
H&E: hematoxylin and eosin
HSP: heat shock protein
ICD: immunogenic cell death
KEGG: Kyoto Encyclopedia of Genes and Genomes
PBS: phosphate‐buffered saline
PCA: principal component analysis
PDOs: patient‐derived organoids
PI: propidium iodide
qRT‐PCR: quantitative real‐time polymerase chain reaction
RMS: root mean square
RNA‐seq: RNA sequencing
SB525334: selective ALK5/TGFβRI inhibitor
SD: standard deviation
SMAD: small mothers against decapentaplegic
TGF‐β: transforming growth factor beta
TGFβRI: transforming growth factor beta receptor I
TMZ: temozolomide
TTF: tumor Treating Fields
WB: Western blotting

## 1 Introduction

Glioblastoma (GBM) is the most prevalent and aggressive malignant tumor of the central nervous system. Although maximal safe resection followed by radiotherapy and temozolomide (TMZ) chemotherapy has improved patient outcomes, treatment efficacy remains limited by tumor heterogeneity, infiltrative growth, and early chemoresistance [1, 2].

Tumor Treating Fields (TTF) is a locoregional biophysical modality that applies low‐intensity, intermediate‐frequency alternating electric fields (1–3 V/cm, 100–300 kHz) to disrupt mitotic processes [3]. By interfering with microtubule dynamics, mitotic spindle assembly, and contractile ring formation, TTF can induce chromosomal missegregation, mitotic arrest, and cell death [4, 5]. TTF has been approved for recurrent and newly diagnosed GBM, and randomized studies have demonstrated survival benefits when TTF is incorporated into GBM treatment [6, 7]. However, the molecular responses induced by TTF, particularly in relation to TMZ‐based therapy, remain incompletely defined.

TMZ remains the backbone of GBM chemotherapy [8], but intrinsic and acquired resistance frequently limits its efficacy, with recurrence commonly occurring after standard chemoradiotherapy [2, 9]. Similarly, TTF alone provides incomplete antitumor activity [10]. Because TMZ induces DNA alkylation and replication stress, whereas TTF disrupts mitotic progression and chromosomal segregation, their combination may enhance GBM growth suppression. Previous studies have shown that TTF induces apoptosis, suppresses migration‐ and invasion‐related phenotypes, and exerts antiproliferative effects [11, 12]. TTF has also been reported to trigger immunogenic cell death and enhance antitumor immune responses [13, 14], but the intracellular signaling programs associated with TTF‐mediated enhancement of TMZ‐related effects remain insufficiently understood.

The TGF‐β/SMAD pathway has context‐dependent functions in GBM biology [15]. It can suppress proliferation and promote apoptosis in certain contexts [16], but is also associated with EMT‐like programs, immune evasion, stemness, and therapy resistance in advanced malignancies [17]. Given its roles in cell‐cycle regulation, DNA damage responses, and apoptosis‐related signaling, SMAD‐associated signaling may contribute to GBM responses to antitumor interventions. However, whether TTF is associated with SMAD‐related signaling changes and whether these changes contribute to apoptosis and growth suppression remain unclear.

In this study, TTF parameters of 200 kHz and 1.75 V/cm RMS and a TMZ concentration of 200 μM were selected based on preclinical evidence [18, 19, 20]. We evaluated TTF alone and with TMZ in GBM cell lines and patient‐derived organoids, explored TTF‐associated molecular signatures by RNA‐seq, and assessed candidate pathways using molecular and functional assays.

## 2 Materials and Methods

### 2.1 Cell Culture and Treatments

U251 GBM cells were obtained from the American Type Culture Collection (ATCC). SHG140 primary GBM cells were established from patient tissue resected at the First Affiliated Hospital of Soochow University [21]. PDOs were generated from GBM samples collected at the Fourth Affiliated Hospital of Soochow University. Cells were cultured in DMEM/F‐12 medium (Gibco, USA; Cat. No. 11320033) supplemented with 10% fetal bovine serum (Gibco, USA; Cat. No. 26140079), penicillin, streptomycin, and gentamicin at 37°C with 5% CO_2_.

Cells were assigned to vehicle control, TMZ, TTF, or TMZ + TTF groups. TMZ was prepared as a 100 mM DMSO stock and diluted to 200 μM immediately before treatment. The final DMSO concentration was 0.2% in all groups. In the TMZ + TTF group, TMZ and TTF were initiated simultaneously, and TMZ was added once without replenishment during the 24–72 h treatment window.

TTF was delivered using the CL‐301A system (An Tai Kang Cheng, Changsha, China). For parameter validation, U251 and SHG140 cells were exposed for 48 h to 100, 200, or 300 kHz at 1.75 V/cm RMS, or to 0.5–3.0 V/cm RMS at 200 kHz. Viability was assessed by CCK‐8 and normalized to non‐TTF controls. Based on these experiments and prior evidence, 200 kHz and 1.75 V/cm RMS were used for subsequent studies. TTF exposure was performed continuously in a 37°C, 5% CO₂ humidified incubator using the device temperature‐control module, with brief interruptions only for imaging or sample collection. In selected experiments, cells were treated with SB525334, a TGFβRI/ALK5 inhibitor (10 μM; Selleck, USA).

ATP‐based viability assays were performed at 24, 48, and 72 h; CCK‐8 assays at 72 h; parameter‐validation assays at 48 h; apoptosis assays at 24 and/or 48 h; Transwell assays at 48 h; wound‐healing and cell‐cycle assays at the indicated time points; and PDO analyses at 0, 24, and 48 h.

### 2.2 Functional Assays

For CCK‐8 assays, cells were seeded in 96‐well plates at 8 × 10^3^–1 × 10^4^ cells/well, treated as indicated, and incubated with 10 μL CCK‐8 reagent (Targetmol, USA; Cat. No. C0005) for 1–2 h at 37°C. Absorbance was measured at 450 nm with 650 nm as reference using a KHB ST‐960 microplate reader. Viability was calculated as [(OD_treatment − OD_blank)/(OD_control − OD_blank)] × 100%. Each group included five replicate wells, which were averaged within each of three independent experiments.

ATP activity was measured using an ATP‐based luminescence assay kit (Arcegen, China; Cat. No. C331309E). After treatment, 100 μL detection reagent was added, and luminescence was recorded using a BioTek Synergy HTX reader and normalized to control values or ATP standards. Five replicate wells per treatment group were averaged within each of three independent experiments.

For colony formation, treated cells were seeded in 6‐well plates at 1000 cells/well and cultured for 14 days. Colonies were fixed with 4% paraformaldehyde, stained with 0.5% crystal violet, and quantified using ImageJ; colonies larger than 0.5 mm were counted.

Transwell migration was assessed using 8‐μm pore chambers (Corning, USA). Treated cells were seeded in serum‐free medium in the upper chamber, with 10% FBS‐containing medium in the lower chamber. After 48 h, migrated cells were fixed, stained with crystal violet, imaged in three random fields per insert, and counted using ImageJ. Field‐level counts were averaged within each independent experiment. For wound‐healing assays, confluent cells were scratched with a sterile 200‐μL pipette tip and imaged at 0, 24, and 48 h using an Olympus IX71 microscope. Wound closure was quantified using ImageJ from three images per treatment group and time point in each independent experiment. Image‐level measurements were averaged before analysis, and the results were interpreted together with the Transwell and viability data.

### 2.3 Apoptosis and Cell‐Cycle Assays

For cleaved caspase‐3 immunofluorescence, cells were fixed with 4% paraformaldehyde, permeabilized with 0.5% Triton X‐100, blocked with 5% BSA, and incubated overnight at 4°C with anti‐cleaved caspase‐3 antibody (Proteintech, USA; Cat. No. 25546–1‐AP; 1:200). Cells were then incubated with Alexa Fluor 594‐conjugated secondary antibody (Invitrogen, USA; Cat. No. A‐11037; 1:500), counterstained with DAPI (1 μg/mL), and imaged using a BZ‐X810 fluorescence microscope (Keyence, Japan). Mean fluorescence intensity was quantified in three random 20× fields per independent experiment using ImageJ and normalized to DAPI. Field‐level measurements were averaged before statistical analysis.

Apoptosis was also measured using an Annexin V‐FITC apoptosis detection kit (Beyotime, China; Cat. No. C1062L). Cells were stained with Annexin V‐FITC and propidium iodide for 15 min and analyzed on a CytoFLEX flow cytometer, with at least 10,000 events acquired per sample in each of three independent experiments. Annexin V^+^/PI^−^ and Annexin V^+^/PI^+^ cells were defined as early and late apoptotic populations, respectively.

For cell‐cycle analysis, cells were fixed in 70% ethanol, treated with RNase A (100 μg/mL), stained with PI (50 μg/mL), and analyzed using a BD FACSCalibur system, with at least 10,000 events acquired per sample. Cell‐cycle distribution was evaluated with ModFit LT 5.0 [22].

### 2.4 Western Blot and qPCR

Total protein was extracted using RIPA buffer with protease and phosphatase inhibitors, quantified by BCA assay, separated by SDS‐PAGE, and transferred to nitrocellulose membranes. Membranes were incubated overnight at 4°C with rabbit anti‐p‐SMAD2 (Selleck, USA; Cat. No. F1689; 1:1000), rabbit anti‐p‐SMAD3 (Selleck, USA; Cat. No. F2846; 1:2000), mouse anti‐SMAD2 (Santa Cruz, USA; Cat. No. sc‐8332; 1:1000), rabbit anti‐TGFβRI (Proteintech, USA; Cat. No. 55391–1‐AP; 1:1000), rabbit anti‐BIM (Selleck, USA; Cat. No. F0222; 1:1000), rabbit anti‐PUMA (Selleck, USA; Cat. No. F0558; 1:1000), rabbit anti‐p‐CHK1 (Selleck, USA; Cat. No. F0242; 1:1000), or mouse anti‐GAPDH (Affinity, China; Cat. No. AF7021; 1:5000). Membranes were then incubated with goat anti‐mouse IgG (Proteintech, USA; Cat. No. SA00001‐1; 1:10,000) or goat anti‐rabbit IgG (ABclonal, China; Cat. No. AS014; 1:10,000). Signals were detected by enhanced chemiluminescence, normalized to loading controls, and quantified using ImageJ [23].

Total RNA was extracted using an RNA extraction kit (Beyotime, China; Cat. No. R0032). Reverse transcription and qPCR were performed using a Beyotime qRT‐PCR kit (Cat. No. D7268M). The PCR program was 95°C for 30 s, followed by 40 cycles of 95°C for 5 s and 60°C for 30 s. *SMAD3*, *SMAD2*, and *TGFβR1* were analyzed, with *GAPDH* as the reference gene. Relative expression was calculated using the 2^−ΔΔCt method [24]. Each biological replicate was analyzed in three technical replicate wells, which were averaged before statistical analysis. Three independent experiments were performed.

### 2.5 RNA‐Seq and Enrichment Analysis

Total RNA from control and TTF‐treated U251 cells (*n* = 3 independent biological replicates per group) was used to explore TTF‐associated transcriptional changes. RNA integrity was confirmed with RIN ≥ 7.0. Poly(A)‐enriched strand‐specific libraries were sequenced on the Illumina platform, generating at least 6 Gb clean data per sample. Reads were trimmed, aligned to GRCh38 using HISAT2, and quantified using featureCounts. Differential expression analysis was performed with DESeq2 using raw counts, with |log_2_FC| > 1 and FDR < 0.05 as thresholds; FPKM values were used only for visualization [25].

GO, KEGG, and Reactome over‐representation analyses were performed using clusterProfiler. Upregulated and downregulated DEGs were analyzed separately, and *p* values were adjusted using the Benjamini–Hochberg method. Terms with adjusted q values < 0.05 were considered significant. Because RNA‐seq included only control and TTF‐treated cells, transcriptomic data were interpreted as exploratory TTF‐associated signatures rather than TMZ+TTF‐specific programs. Candidate pathways were further evaluated in four treatment groups using molecular and functional assays.

### 2.6 PDO Culture and Treatment

GBM PDOs were established from surgically resected and pathologically confirmed specimens obtained at the Fourth Affiliated Hospital of Soochow University. Fresh tumor tissues were processed within 2 h, dissected into 0.5–1 mm^3^ fragments, and cultured in low‐adhesion plates using DMEM/F12 and Neurobasal medium at a 1:1 ratio supplemented with N2, B27 without vitamin A, NEAA, GlutaMAX, insulin, a three‐antibiotic combination, and 2‐mercaptoethanol as previously described. Organoids were maintained at 37°C with 5% CO_2_ for approximately 2 weeks before treatment. Three independent PDO lines derived from three patients were maintained and analyzed separately, and their available clinicopathological and molecular characteristics are summarized in Table 1. Within each PDO line, three organoids with comparable baseline size and morphology were selected for each treatment group.

**TABLE 1:** Clinicopathological and molecular characteristics of patients from whom glioblastoma patient‐derived organoid lines were established.

PDO line	Age (years)	Sex	Tumor status	Histopathological diagnosis	WHO grade	IDH status	Ki‐67 index (%)
PDO‐1	64	Female	Primary	Glioblastoma	4	IDH‐wildtype	60
PDO‐2	57	Female	Recurrent	Glioblastoma	4	IDH‐wildtype	60
PDO‐3	36	Male	Recurrent	Glioblastoma	4	IDH‐wildtype	Not available

Abbreviations: IDH, isocitrate dehydrogenase; PDO, patient‐derived organoid; WHO, World Health Organization.

PDOs were assigned to vehicle control, TMZ, TTF, and TMZ+TTF groups. TMZ was diluted to 200 μM in organoid medium, with 0.2% DMSO matched in all groups. TTF was applied at 200 kHz and 1.75 V/cm RMS using the CL‐301A system. TMZ and TTF were initiated simultaneously in the combination group, and TMZ was not replenished during the 0–48 h PDO treatment window. Organoids were harvested at 0, 24, and 48 h.

After fixation, paraffin embedding, and H&E staining, organoid morphology was examined at 10× and 40× magnification. Immunofluorescence staining for cleaved caspase‐3 (Proteintech, USA; Cat. No. 25128–1‐AP) and Ki‐67 (Cell Signaling Technology, USA; Cat. No. 9449S) was performed to assess apoptosis and proliferation. For immunofluorescence analysis, one image was acquired from each organoid and quantified using ImageJ. The three organoid‐level measurements within each PDO line and treatment group were averaged to generate one PDO‐line‐level value.

### 2.7 Statistical Analysis

Experiments were independently repeated three times unless otherwise stated, and data are presented as mean ± SD. Reported n values denote independent biological or experimental replicates. Technical subsamples, including wells, images, and organoids, were averaged within each replicate; flow‐cytometric events were not treated as independent replicates. TTF parameter‐validation assays were analyzed using one‐way ANOVA followed by Dunnett's test. ATP‐based time‐course assays were analyzed using two‐way ANOVA followed by Tukey's test, whereas wound‐healing and cell‐cycle assays were analyzed using two‐way ANOVA followed by Sidak's test. Other multiple‐group comparisons were analyzed using one‐way ANOVA followed by Tukey's test. For PDO analyses, the independent PDO line was defined as the biological replicate and statistical unit (*n* = 3), whereas individual organoids and images were considered technical subsamples and averaged within each PDO line before statistical analysis. PDO immunofluorescence data were analyzed using repeated‐measures one‐way ANOVA followed by Tukey's test, with the PDO line treated as the matched replicate. Exact n values are specified in the corresponding figure legends. Significance was defined as *p < 0.05, **p < 0.01, ***p < 0.001, and ****p < 0.0001.

## 3 Results

### 3.1 TTF Parameter Validation and TMZ+TTF‐Mediated Suppression of GBM Cell Growth and Migration‐Related Phenotypes

TTF parameter‐validation assays were first performed in U251 and SHG140 cells. Under a fixed field intensity of 1.75 V/cm RMS, 200 kHz produced stable inhibition of cell viability in both models, whereas 300 kHz did not further enhance this effect. Under a fixed frequency of 200 kHz, increasing field intensity progressively reduced viability, with reproducible inhibition within the 1.5–2.0 V/cm RMS range. Therefore, 200 kHz and 1.75 V/cm RMS were selected for subsequent experiments (Figure S1A,B).

The effects of TMZ, TTF, and TMZ+TTF were then evaluated using viability, colony formation, and migration assays. ATP‐based luminescence assays showed time‐dependent reductions in ATP activity after treatment, with the strongest decrease in the TMZ+TTF group at 48 and 72 h (Figure 1A,B). CCK‐8 assays at 72 h similarly showed that TMZ+TTF reduced cell viability more strongly than either single treatment, although the relative effects of TMZ and TTF monotherapy varied between U251 and SHG140 cells (Figure 1C,D). Colony formation assays further showed that TMZ or TTF alone reduced clonogenic growth, whereas TMZ+TTF produced the most pronounced suppression (Figure 1E,F).

**FIGURE 1:**
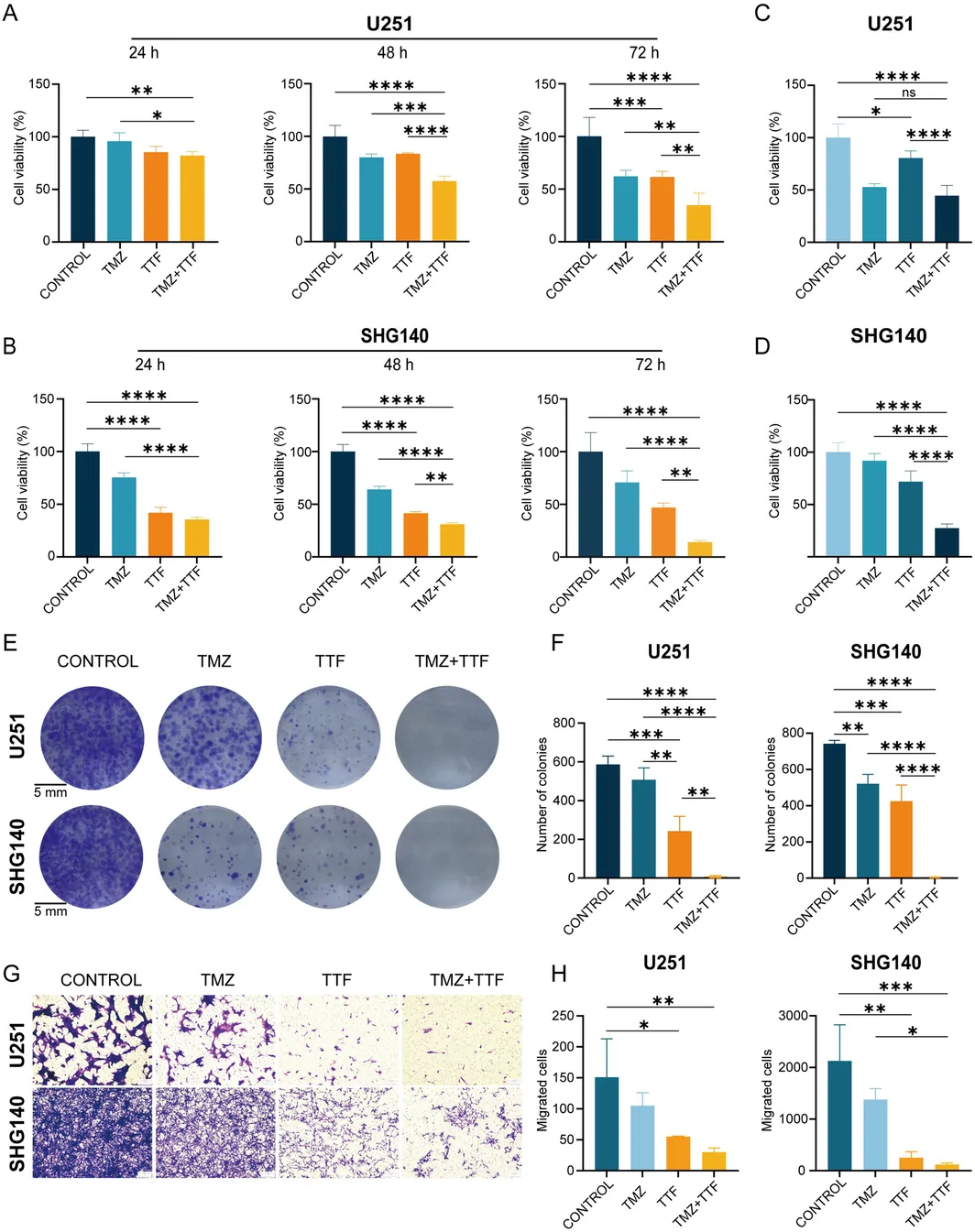
Combined TMZ+TTF treatment produces greater growth suppression, clonogenic inhibition, and reduction in migratory capacity in glioblastoma cells. (A, B) ATP‐based luminescence viability assays showing relative ATP activity in U251 and SHG140 cells at 24, 48, and 72 h under different treatment conditions. (C, D) CCK‐8 viability assays of U251 and SHG140 cells at 72 h. (E) Representative colony formation images of cells subjected to different treatments; scale bar = 5 mm. (F) Quantitative analysis of colony numbers across groups. (G) Transwell migration assays showing migrated cells under different treatment conditions using crystal violet staining; scale bar = 500 μm. (H) Quantification of migrated cells. Vehicle control indicates complete culture medium containing 0.2% DMSO, matching the final DMSO concentration in TMZ‐containing groups. Pairwise comparisons among treatment groups were performed for the quantitative analyses, including TMZ versus TMZ+TTF and TTF versus TMZ+TTF comparisons in the Transwell migration assay. For ATP‐based and CCK‐8 assays, five replicate wells were averaged within each independent experiment. For Transwell analysis, three random fields per insert were averaged to generate one value per experiment. Data are presented as mean ± SD (*n* = 3 independent experiments). Statistical analysis for ATP‐based time‐course viability assays was performed using two‐way ANOVA followed by Tukey's multiple comparisons test. Statistical analysis for CCK‐8, colony formation, and Transwell migration assays was performed using one‐way ANOVA followed by Tukey's multiple comparisons test. *p < 0.05, **p < 0.01, ***p < 0.001, ****p < 0.0001.

Transwell assays showed reduced migrated‐cell numbers after TMZ or TTF monotherapy and a further reduction after TMZ+TTF treatment (Figure 1G,H). Wound‐healing assays also showed decreased wound closure after treatment, particularly in the TMZ+TTF group at 48 h (Figure S1C,D). Because wound closure can be influenced by migration, proliferation, and survival, these results were interpreted as supportive evidence together with Transwell and viability assays. Overall, combined TMZ+TTF treatment produced greater inhibition of GBM cell growth, clonogenicity, and migration‐related phenotypes than either monotherapy, supporting an enhanced combined effect without establishing pharmacological synergy.

### 3.2 Combined TMZ+TTF Treatment Enhances Apoptotic Responses in GBM Cells

Cleaved caspase‐3 immunofluorescence was used to evaluate apoptosis‐related responses. Vehicle‐treated cells showed weak cleaved caspase‐3 signals, whereas TMZ or TTF monotherapy increased cleaved caspase‐3 staining. The TMZ+TTF group showed the strongest cleaved caspase‐3 activation in both U251 and SHG140 cells (Figure 2A,B).

**FIGURE 2:**
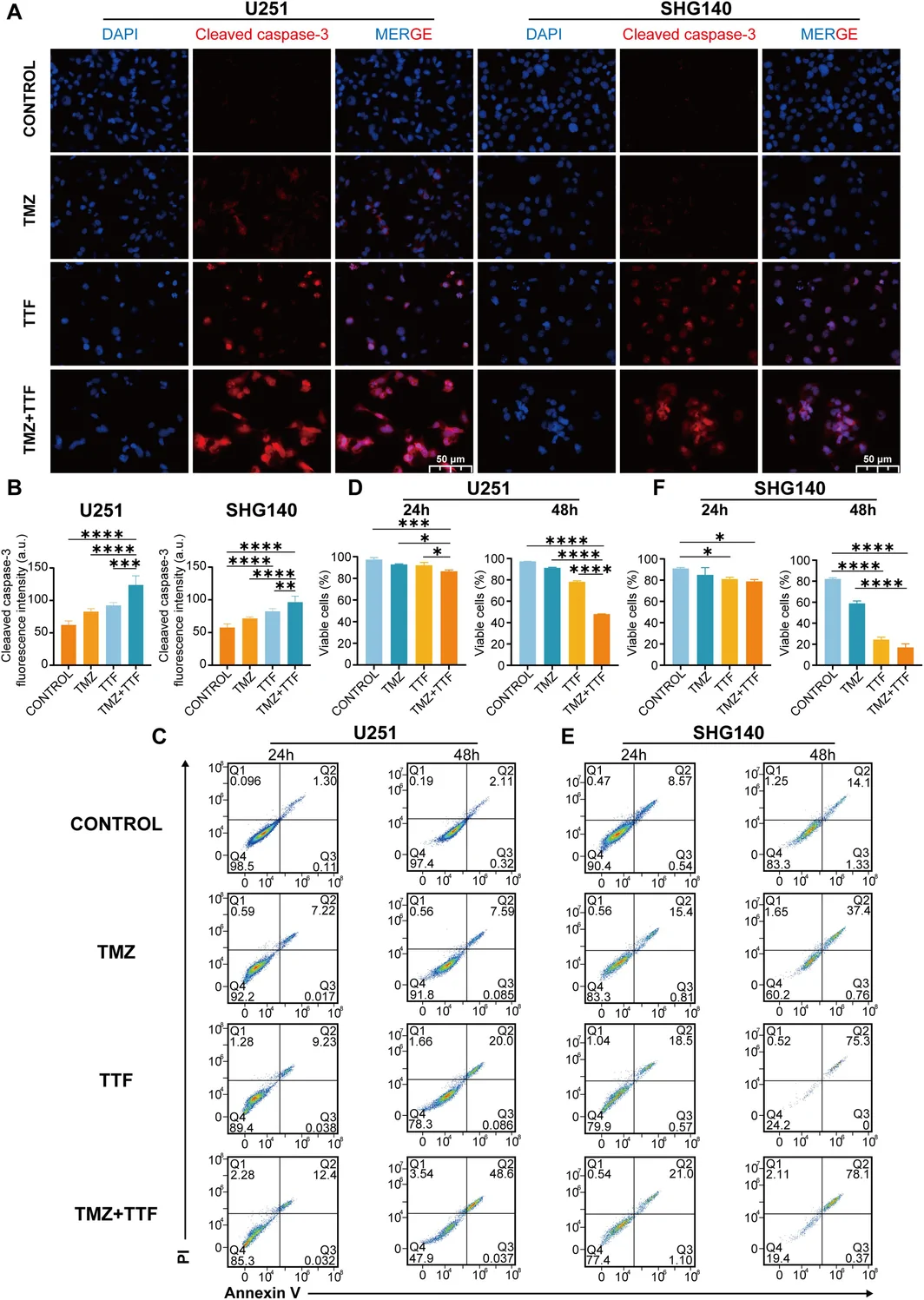
TMZ+TTF treatment enhances apoptotic responses in glioblastoma cells. (A) Immunofluorescence staining of cleaved caspase‐3 (red) and nuclei (blue, DAPI) in U251 and SHG140 cells under different treatment conditions. (B) Quantification of cleaved caspase‐3 fluorescence intensity. (C, E) Representative Annexin V‐FITC/PI flow cytometry plots showing the distribution of viable and apoptotic cell populations at 24 and 48 h in U251 and SHG140 cells, respectively. Lower left: Viable cells; lower right: Early apoptotic cells; upper right: Late apoptotic cells; upper left: PI‐positive non‐apoptotic/dead cells. (D, F) Quantitative analysis of viable‐cell fractions derived from Annexin V‐FITC/PI flow cytometry in U251 and SHG140 cells at 24 and 48 h. For cleaved caspase‐3 quantification, three random 20× fields were averaged within each independent experiment. At least 10,000 events were acquired per flow‐cytometry sample. Data are presented as mean ± SD (*n* = 3 independent experiments). Statistical analysis was performed using one‐way ANOVA followed by Tukey's multiple comparisons test within each time point. *p < 0.05, **p < 0.01, ***p < 0.001, ****p < 0.0001.

Consistently, Annexin V‐FITC/PI flow cytometry showed that TMZ or TTF increased apoptotic cell populations and reduced the viable‐cell fraction, while TMZ+TTF produced the greatest reduction in the viable‐cell fraction (Figure 2C–F). At 48 h, the TMZ+TTF group showed a lower viable‐cell fraction than at 24 h, accompanied by increased Annexin V‐positive apoptotic populations. These findings indicate that combined TMZ+TTF treatment produced a greater apoptosis‐associated response than either treatment alone, consistent with an enhanced combined effect in GBM cells.

### 3.3 Exploratory RNA‐Seq Identifies TTF‐Associated Transcriptomic Changes in U251 GBM Cells

RNA‐seq was performed in control and TTF‐treated U251 cells to explore transcriptional changes associated with TTF exposure. Quality‐control metrics showed Q20/Q30 values > 95%, appropriate GC content, mapping rates > 95%, and unique alignment > 93%. Biological replicates showed strong concordance, and PCA separated control and TTF‐treated samples, indicating distinct transcriptomic profiles after TTF exposure (Figure S2A,B).

Using |log_2_FC| > 1 and FDR < 0.05, 4145 differentially expressed genes were identified, including 2578 upregulated and 1567 downregulated genes (Figure S2C). Upregulated genes included stress‐response genes such as HSPA6, HSPA1A, and HSPA1B. GO, KEGG, and Reactome enrichment analyses indicated that TTF exposure was associated with transcriptional regulation, chromatin‐related processes, DNA replication, and cell‐cycle pathways (Figure S2D,E). Because RNA‐seq was performed only in control and TTF‐treated cells, these data were interpreted as exploratory TTF‐associated molecular signatures rather than transcriptional programs specific to TMZ+TTF treatment. Candidate pathways were therefore further examined in four treatment groups using molecular and functional assays.

### 3.4 TTF Is Associated With Selected SMAD‐Related Signaling Changes in Four Treatment Groups

KEGG enrichment analysis suggested that TGF‐β/SMAD‐related terms were enriched among TTF‐associated differentially expressed genes (Figure 3A). Because this pathway includes both activating mediators and feedback regulators, enrichment alone was not considered sufficient to establish global pathway activation. Instead, these findings were interpreted as indicating a candidate SMAD‐associated response to TTF exposure.

**FIGURE 3:**
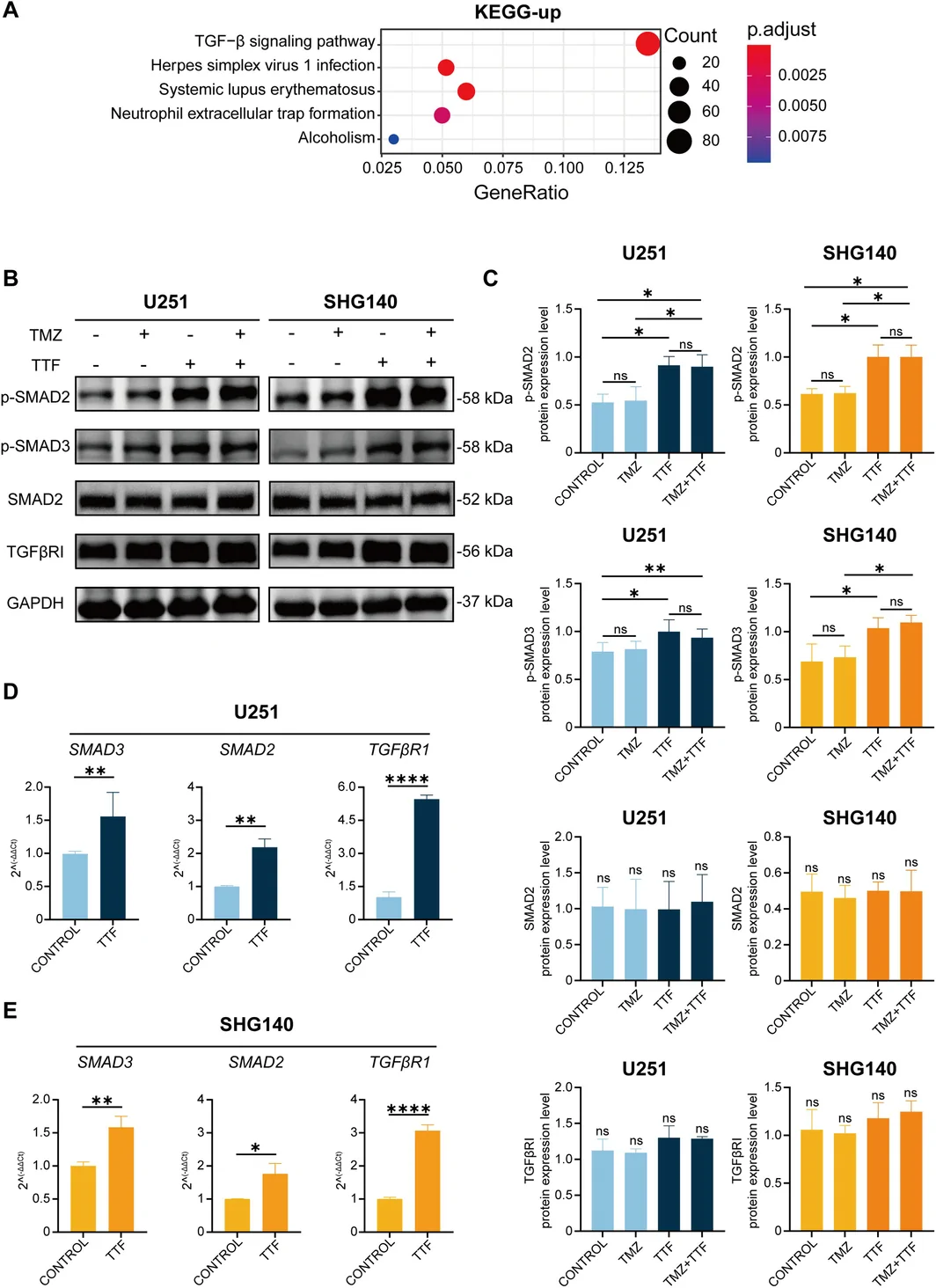
TTF is associated with selected SMAD‐related signaling changes in U251 and SHG140 glioblastoma cells. (A) KEGG enrichment analysis of TTF‐associated differentially expressed genes and heatmap visualization of representative TGF‐β/SMAD‐related genes based on control and TTF‐treated U251 RNA‐seq data. (B, C) Western blot and quantitative analysis of phosphorylated SMAD2 (p‐SMAD2), phosphorylated SMAD3 (p‐SMAD3), total SMAD2, and TGFβRI protein expression in vehicle control, TMZ, TTF, and TMZ+TTF groups. (D, E) qRT‐PCR analysis of *TGFβR1*, *SMAD2*, and *SMAD3* mRNA levels in U251 and SHG140 cells under the indicated treatment conditions. Densitometric values for p‐SMAD2, p‐SMAD3, SMAD2, and TGFβRI were normalized to GAPDH. Data are presented as mean ± SD (*n* = 3 independent experiments). For qRT‐PCR, three technical replicate wells were averaged within each independent experiment. Statistical significance was evaluated using one‐way ANOVA followed by Tukey's multiple comparisons test. *p < 0.05, **p < 0.01, ***p < 0.001, ****p < 0.0001.

Western blot analysis in four treatment groups showed that TTF increased SMAD2 and SMAD3 phosphorylation, whereas TMZ alone had a relatively limited effect. SMAD2/3 phosphorylation in the TMZ+TTF group was comparable to that in the TTF group, suggesting that this response was mainly associated with TTF exposure rather than further enhanced by TMZ (Figure 3B,C). qRT‐PCR analysis showed increased *TGFβR1* and *SMAD3* mRNA expression after TTF treatment, while *SMAD2* showed a mild upward trend (Figure 3D,E). These results indicate that TTF is associated with selected SMAD‐related signaling changes in GBM cells.

### 3.5 TGFβRI/ALK5 Inhibition Partially Attenuates TTF‐Associated SMAD Phosphorylation, Growth Suppression, and Apoptotic Responses

To assess whether SMAD‐associated signaling contributed to TTF‐related effects, cells were treated with the TGFβRI/ALK5 inhibitor SB525334. Western blotting showed that SB525334 markedly attenuated TTF‐induced SMAD2 and SMAD3 phosphorylation (Figure 4A,B).

**FIGURE 4:**
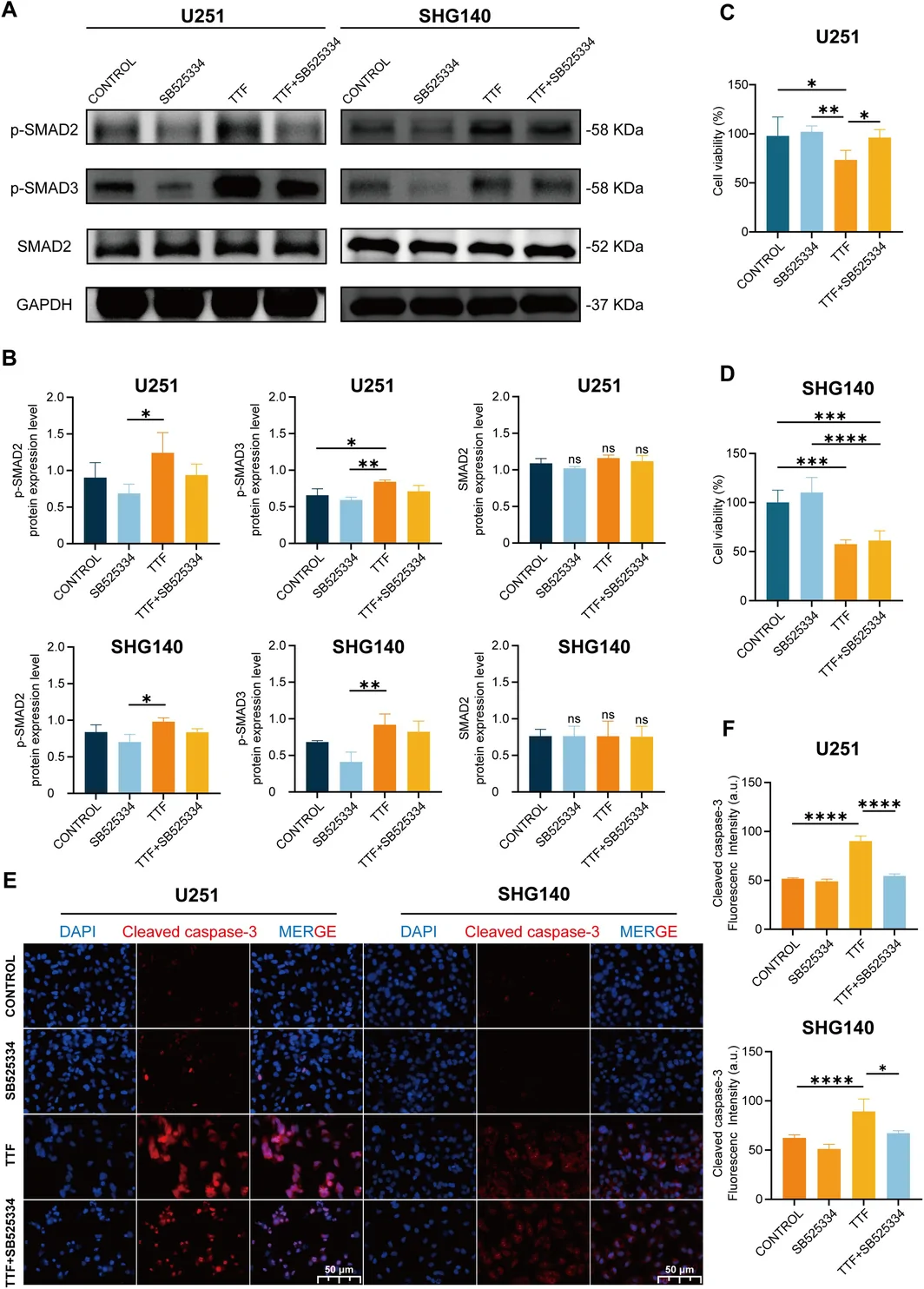
ALK5/TGFβRI inhibition partially attenuates TTF‐associated SMAD phosphorylation, growth suppression, and cleaved caspase‐3 induction. (A) Western blot analysis of phosphorylated SMAD2 (p‐SMAD2), phosphorylated SMAD3 (p‐SMAD3), and total SMAD2 in U251 and SHG140 cells treated with vehicle control, TTF, SB525334, or TTF+SB525334. (B) Quantification of p‐SMAD2, p‐SMAD3, and total SMAD2 protein expression levels. Densitometric values for p‐SMAD2, p‐SMAD3, and SMAD2 were normalized to GAPDH. (C, D) CCK‐8 assays measuring cell viability in U251 and SHG140 cells treated with vehicle control, TTF, SB525334, or TTF+SB525334 under the same 72 h assay conditions used in Figure 1C,D. SB525334 was used at 10 μM as a selective ALK5/TGFβRI inhibitor. (E) Immunofluorescence staining of cleaved caspase‐3 (red) and nuclei (blue, DAPI) in U251 and SHG140 cells under the indicated treatment conditions; merged images are shown. (F) Quantification of cleaved caspase‐3 fluorescence intensity. For cleaved caspase‐3 quantification, three random 20× fields were averaged within each independent experiment. Data are presented as mean ± SD (*n* = 3 independent experiments). Statistical analysis was performed using one‐way ANOVA followed by Tukey's multiple comparisons test. *p < 0.05, **p < 0.01, ***p < 0.001, ****p < 0.0001.

CCK‐8 assays showed that TTF reduced cell viability, whereas SB525334 partially rescued TTF‐associated viability loss in both U251 and SHG140 cells (Figure 4C,D). However, viability was not fully restored to control levels. Cleaved caspase‐3 immunofluorescence further showed that SB525334 reduced TTF‐associated cleaved caspase‐3 activation (Figure 4E,F). These findings suggest that SMAD‐associated signaling may contribute to TTF‐related growth suppression and apoptosis but do not fully account for the overall antitumor effect of TTF.

### 3.6 TTF Is Associated With Downregulation of DNA Replication and Mitotic Pathways and G2/M‐Phase Accumulation

Transcriptomic analyses showed that TTF affected pathways related to DNA replication and cell division. Downregulated genes were enriched in biological processes associated with DNA repair, replication initiation, chromatin‐associated mitosis, DNA strand elongation, and G1/S transition (Figure S3A–E).

Flow cytometry confirmed that TTF increased the proportion of cells in G2/M phase and reduced the S‐phase fraction at 24 and 48 h, consistent with the G2/M arrest (Figure S3F,G). Western blotting further showed decreased p‐CHK1 expression and increased BIM and PUMA expression after TTF exposure (Figure S4). These results suggest that TTF impairs DNA replication and mitotic progression and is accompanied by altered checkpoint signaling and enhanced proapoptotic signaling.

### 3.7 Combined TMZ+TTF Treatment Enhances Structural Disruption and Apoptosis‐Associated Responses in GBM Organoids

Three independent GBM PDO lines, each derived from a different patient and analyzed separately, were used to assess treatment‐associated effects in a three‐dimensional ex vivo model. Vehicle control organoids largely maintained spherical architecture during the observation period, with partial loosening by 48 h. TMZ‐treated organoids showed cell shedding and vacuole formation, while TTF‐treated organoids displayed progressive disorganization. More pronounced structural disruption was observed in the TMZ+TTF group as early as 24 h, with greater loss of structural integrity by 48 h than with either monotherapy (Figure 5).

**FIGURE 5:**
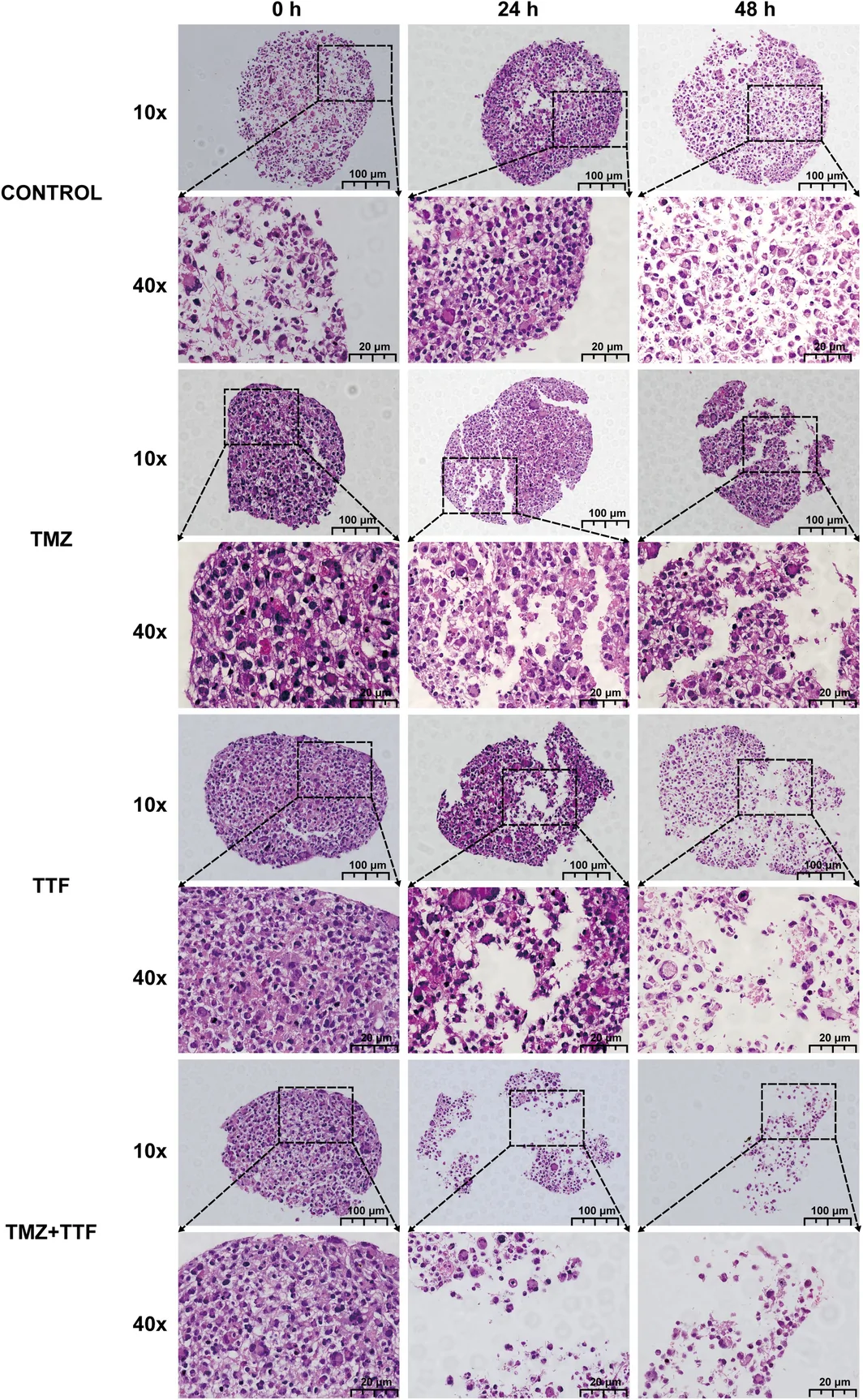
Combined TMZ+TTF treatment is associated with greater morphological disruption in GBM patient‐derived organoids. Representative H&E‐stained images of GBM patient‐derived organoids treated with vehicle control, TMZ, TTF, or TMZ+TTF for 0, 24, and 48 h and examined at 10× and 40× magnification. Vehicle control organoids largely maintained their structural integrity up to 48 h, whereas TMZ or TTF monotherapy induced partial structural disruption at 24 h and more evident disintegration by 48 h. The TMZ+TTF group showed more pronounced structural disruption at 24 h and greater loss of structural integrity by 48 h than either monotherapy. Representative images were obtained from three independent PDO lines derived from three patients, and H&E staining was used for qualitative morphological assessment. Vehicle control indicates organoid culture medium containing 0.2% DMSO, matching the final DMSO concentration in TMZ‐containing groups. Scale bars: 100 μm at 10× and 20 μm at 40×.

Using the independent PDO line as the statistical unit, quantitative immunofluorescence analysis showed increased cleaved caspase‐3 fluorescence and reduced Ki‐67 fluorescence in TTF‐exposed PDOs, with the greatest changes observed after combined TMZ+TTF treatment (Figure S5). Together with the qualitative H&E findings, these results suggest increased apoptosis‐associated responses, reduced proliferation‐associated marker expression, and more pronounced morphological disruption after combined treatment.

## 4 Discussion

In this study, TTF suppressed GBM cell growth and enhanced TMZ‐associated reductions in viability, clonogenic growth, and migration‐related phenotypes, while increasing apoptosis‐associated responses. Compared with either monotherapy, TMZ+TTF produced greater inhibitory effects across functional assays, consistent with an enhanced combined effect and supporting further evaluation of TTF as a complementary modality to TMZ. However, because TMZ‐resistant GBM models were not included, whether this strategy can overcome established TMZ resistance remains to be determined [7, 26].

The biological rationale for combining TMZ with TTF is based on their distinct but potentially complementary mechanisms. TMZ primarily induces DNA alkylation and replication‐associated stress, whereas TTF interferes with mitotic progression and chromosomal segregation, thereby promoting mitotic stress and cell death [27, 28, 29, 30]. In our models, combined TMZ+TTF treatment produced greater cleaved caspase‐3 activation and a lower viable‐cell fraction than either treatment alone. However, the present study used a single TMZ concentration under fixed TTF conditions and did not include a TMZ dose–response matrix or formal interaction analyses, such as Bliss, Loewe, highest single agent, or Chou–Talalay modeling. Therefore, the results support an enhanced combined effect but do not establish pharmacological synergy or a formal drug–treatment interaction.

Exploratory RNA‐seq analysis showed that TTF exposure was associated with changes in transcriptional regulation, chromatin‐associated processes, DNA replication, cell‐cycle pathways, and stress‐response genes, including HSP family members. Because RNA‐seq was performed only in control and TTF‐treated U251 cells, these data represent exploratory TTF‐associated molecular signatures rather than TMZ+TTF‐specific transcriptional programs and do not establish direct causality or combination‐specific responses.

Among the candidate pathways, SMAD‐related signaling was further evaluated because TGF‐β/SMAD‐related terms were enriched among TTF‐associated differentially expressed genes. This enrichment was not interpreted as definitive activation of the entire TGF‐β pathway, because the pathway includes positive mediators and negative‐feedback regulators and has context‐dependent effects in cancer [31, 32]. Western blotting showed increased SMAD2/3 phosphorylation after TTF exposure, whereas TMZ alone had a limited effect, and TMZ+TTF showed phosphorylation levels comparable to TTF alone. Thus, this SMAD‐associated response was primarily associated with TTF exposure and was not further increased by TMZ.

The interpretation of SMAD‐related signaling in GBM requires caution. TGF‐β/SMAD signaling can induce cell‐cycle arrest and apoptosis in certain contexts, but in advanced malignancies, including GBM, it is also associated with invasion, immune evasion, stemness maintenance, therapeutic resistance, and stromal remodeling [33, 34, 35, 36]. Therefore, increased SMAD2/3 phosphorylation should not be interpreted as global activation of a tumor‐suppressive TGF‐β program. Instead, TTF exposure was associated with selected SMAD‐related changes that may contribute to apoptosis‐associated and growth‐inhibitory responses under the present in vitro conditions.

Pharmacological inhibition with SB525334 was consistent with a contributory role for SMAD‐associated signaling. SB525334 attenuated TTF‐associated SMAD2/3 phosphorylation and partially reduced TTF‐related growth suppression and cleaved caspase‐3 induction. However, this rescue was incomplete, and viability was not restored to vehicle control levels. Thus, SMAD‐associated signaling may contribute to, but does not appear to fully account for, TTF‐related effects. Additional mechanisms, including mitotic disruption, DNA replication stress, and broader stress‐response pathways, may also be involved.

Consistently, transcriptomic and flow cytometric analyses indicated that TTF was associated with alterations in DNA replication and cell‐cycle progression. Downregulated genes were enriched in DNA repair, replication initiation, DNA strand elongation, and G1/S transition pathways, while flow cytometry demonstrated G2/M‐phase accumulation after TTF treatment. These results are consistent with impaired DNA replication and mitotic progression accompanying TTF‐associated growth suppression, but do not establish a direct causal pathway [37, 38]. Western blotting further showed decreased p‐CHK1 and increased BIM and PUMA expression, changes consistent with altered checkpoint and apoptosis‐associated signaling. Further studies using additional DNA damage markers and mechanistic rescue approaches are needed to clarify the causal relationships among these changes.

GBM PDOs were used to evaluate whether the in vitro findings could be observed in a more tissue‐like context [39]. Vehicle control organoids largely maintained structural integrity, whereas TMZ or TTF monotherapy induced partial disruption. PDOs exposed to TMZ+TTF showed more pronounced structural disruption and loss of structural integrity by 48 h. However, these H&E‐based findings were qualitative, as organoid area, circularity, direct viability, and a predefined collapse score were not systematically quantified. Increased cleaved caspase‐3 signals and reduced Ki‐67 expression in TTF‐exposed organoids further supported apoptosis‐associated responses and reduced proliferative activity. Nevertheless, PDOs do not fully recapitulate the in vivo tumor microenvironment, immune contexture, vascular architecture, blood–brain barrier, or tissue‐level electric‐field distribution [40].

Several limitations should be acknowledged. First, this study used TMZ‐sensitive GBM models; therefore, whether TTF enhances TMZ responses in TMZ‐resistant or recurrent GBM remains unresolved [41]. Second, RNA‐seq was limited to control and TTF‐treated cells, and the single‐dose design without formal interaction modeling precluded characterization of combination‐specific signatures and formal assessment of pharmacological interaction. Third, normal neural cell types were not included, so therapeutic selectivity cannot be directly assessed [7]. Fourth, no in vivo GBM model was used. In vivo validation would strengthen translational relevance by assessing tumor growth, survival, tissue‐level apoptosis and proliferation, pharmacodynamic responses, and microenvironmental effects [42]. However, the TTF system used here was designed for controlled in vitro and ex vivo exposure and was not technically compatible with stable and reproducible electric‐field delivery in live animals [43]. Future studies will require animal‐compatible TTF platforms with dedicated electrode configurations, stable field control, temperature monitoring, and appropriate immobilization or implantation strategies [44].

Overall, TTF‐associated growth suppression was accompanied by alterations in DNA replication and cell‐cycle progression, G2/M‐phase accumulation, apoptosis‐associated responses, and selected SMAD‐related signaling changes. Combined TMZ+TTF treatment produced greater antitumor effects than either monotherapy in GBM cell lines and PDO models, supporting further evaluation of TTF as a complementary modality to TMZ. However, these findings remain preclinical in vitro and ex vivo evidence rather than definitive in vivo therapeutic proof.

## 5 Conclusion

This study shows that TTF enhances TMZ‐associated growth suppression and apoptosis‐related responses in GBM cell lines and patient‐derived organoids. These effects were accompanied by DNA replication and cell‐cycle blockade, G2/M‐phase accumulation, proapoptotic signaling, and selected SMAD‐related signaling changes. Partial attenuation by TGFβRI/ALK5 inhibition suggests that SMAD‐associated signaling may contribute to, but does not fully explain, TTF‐related biological responses. Overall, these findings support TTF as a complementary modality that enhances TMZ‐associated antitumor effects in GBM models, while formal pharmacological interaction remains to be determined.

## Author Contributions

Haiping Zhu and Yingbo Hou contributed equally to this work and share first authorship. Yingbo Hou, Haiping Zhu, and Yulun Huang were responsible for conceptualization and supervision of the study. Haiping Zhu, Yingbo Hou, Xuetao Li, and Yi Tang designed experiments. Zuoyu Jiang, Yang Zhang, Xuewen Zhang, Jiaming Du, Yang Zhu, Hanmiao Dong, Yeyang Xu, Ruoyu Sun, Zijian Han, and Chenyang Li performed experiments and collected data. Jian Lu and Lingzhi Xiong contributed to technical support and provided key experimental materials. Data analysis and interpretation were conducted by Haiping Zhu, Yingbo Hou, and Xuetao Li. Haiping Zhu and Yingbo Hou drafted the initial manuscript. Yulun Huang revised and finalized the manuscript. All authors read and approved the final version of the manuscript.

## Funding

This work was supported by the National Natural Science Foundation of China, 82173279, 82472981. The National Science and Technology Resource Sharing Service Platform Project, YCZYPT [2020]06‐1. The Suzhou Science and Technology Development Plan Project, SZM2022017. The Suzhou Municipal Health Commission Scientific Research Project, LCZX202134. The Suzhou Medical and Health Innovation Project, CXYJ2024A05. The Suzhou Industrial Park Healthcare Talent Support Initiative, 2024–54. The Gusu Talent Program, 2024‐105.

## Ethics Statement

The collection and research use of human glioblastoma tissues and the generation of patient‐derived organoids were approved by the Ethics Committee of the Fourth Affiliated Hospital of Soochow University (Approval No. 242175). The study was conducted in accordance with the Declaration of Helsinki.

## Consent

Written informed consent was obtained from all participants before tissue collection.

## Conflicts of Interest

Jian Lu and Lingzhi Xiong are affiliated with Hunan An Tai Kang Cheng Biotechnology Co. Ltd., which provided the CL‐301A TTF system used in this study. The company‐affiliated authors also provided technical support and key experimental materials related to the TTF system. These relationships represent potential financial and/or non‐financial competing interests. Except for the relationships disclosed above, the authors declare no other competing interests.

## Supporting information


**Figure S1:** TTF parameter validation and wound‐healing analysis in GBM cells. (A) Frequency‐response validation of TTF in U251 and SHG140 cells exposed to 100, 200, or 300 kHz at a fixed field intensity of 1.75 V/cm RMS for 48 h. Relative cell viability was assessed using the CCK‐8 assay and normalized to the corresponding non‐TTF control group. (B) Intensity‐response validation of TTF in U251 and SHG140 cells exposed to 200 kHz TTF at 0.5–3.0 V/cm RMS for 48 h. Relative cell viability was assessed using the CCK‐8 assay and normalized to the corresponding non‐TTF control group. (C) Representative wound‐healing images of U251 and SHG140 cells treated with vehicle control, TMZ, TTF, or TMZ + TTF at 0, 24, and 48 h. (D) Quantitative analysis of wound closure percentage measured using ImageJ. Because wound closure can be influenced by cell migration, proliferation, and treatment‐related changes in cell survival, these results were interpreted as supportive evidence of migration‐related changes. Five replicate wells were averaged within each parameter‐validation experiment. For wound‐healing analysis, three images per treatment group and time point were averaged within each independent experiment. Data are presented as mean ± SD (*n* = 3 independent experiments). Parameter‐validation assays were analyzed using one‐way ANOVA followed by Dunnett's multiple comparisons test within each cell line. Wound‐closure data were analyzed using two‐way ANOVA followed by Sidak's multiple comparisons test. *p < 0.05, **p < 0.01, ***p < 0.001, ****p < 0.0001.
**Figure S2:** TTF treatment alters the transcriptomic landscape of U251 glioblastoma cells. (A) Boxplot showing the distribution of gene expression levels between vehicle control and TTF‐treated U251 cells. (B) Principal component analysis (PCA) based on variance‐stabilized counts from DESeq2, showing separation between vehicle control and TTF‐treated samples and clustering of biological replicates within each group. (C) Volcano plot of differentially expressed genes between vehicle control and TTF‐treated U251 cells. Upregulated genes are shown in red, downregulated genes in blue, and non‐significant genes ingray. (D) GO enrichment analysis of differentially expressed genes categorized into biological process (BP), cellular component (CC), and molecular function (MF). (E) KEGG and Reactome enrichment analyses showing representative pathways associated with chromatin‐related processes, transcriptional regulation, DNA replication, and cell‐cycle regulation. Differentially expressed genes were defined using thresholds of |log₂FC| > 1 and false discovery rate (FDR) < 0.05. RNA‐seq was performed using three independent biological replicates per group.
**Figure S3:** TTF treatment is associated with downregulation of DNA replication and cell‐cycle pathways and G2/M‐phase accumulation. (A–C) GO enrichment analyses of downregulated genes in the biological process (BP), cellular component (CC), and molecular function (MF) categories. (D) Reactome enrichment analysis showing downregulated pathways related to DNA replication, DNA strand elongation, G1/S transition, and related biological processes. (E) KEGG pathway enrichment analysis showing downregulated pathways associated with DNA replication and cell‐cycle regulation. (F) Representative flow cytometry histograms showing cell‐cycle distribution in control and TTF‐treated cells at 24 h and 48 h. (G) Quantitative analysis of cell‐cycle phase distribution, showing increased G2/M‐phase accumulation after TTF treatment. At least 10,000 events were acquired per sample in each of three independent experiments. Data are presented as mean ± SD (*n* = 3 independent experiments). Statistical analysis was performed using two‐way ANOVA followed by Sidak's multiple comparisons test. *p < 0.05, **p < 0.01, ***p < 0.001, ****p < 0.0001.
**Figure S4:** Western blot analysis of DNA damage checkpoint‐ and apoptosis‐related markers in glioblastoma cells. (A) Representative Western blots of phosphorylated CHK1 (p‐CHK1), BIM, and PUMA in U251 and SHG140 cells treated with vehicle control, TMZ, TTF, or TMZ + TTF. GAPDH served as the loading control for p‐CHK1, and β‐tubulin served as the loading control for BIM and PUMA. (B, C) Quantitative analysis of protein expression levels in U251 and SHG140 cells, respectively. Data are presented as mean ± SD (*n* = 3 independent experiments). Statistical significance was determined using one‐way ANOVA followed by Tukey's multiple comparisons test. *p < 0.05, **p < 0.01, ***p < 0.001; ns, not significant.
**Figure S5:** Immunofluorescence analysis of apoptosis‐ and proliferation‐related markers in GBM patient‐derived organoids under different treatments. (A, B) Representative immunofluorescence images of cleaved caspase‐3 (red) and quantitative analysis of fluorescence intensity in vehicle control, TMZ, TTF, and TMZ + TTF groups. (C, D) Representative immunofluorescence images of Ki‐67 (green) and quantitative analysis of fluorescence intensity across the same treatment groups. Nuclei were counterstained with DAPI (blue). Scale bars: 100 μm; the same scale applies to all fluorescence images within each panel. Data are presented as mean ± SD from three independent PDO lines derived from three patients (*n* = 3 biological replicates). For each PDO line and treatment group, three organoids were analyzed, and one fluorescence image was quantified per organoid. The three organoid‐level measurements were averaged to generate one PDO‐line‐level value before statistical analysis. Individual organoids and images were considered technical subsamples rather than independent biological replicates. Statistical analysis was performed using repeated‐measures one‐way ANOVA followed by Tukey's multiple comparisons test, with PDO line treated as the matched biological replicate. **p < 0.01, ***p < 0.001, ****p < 0.0001; ns, not significant.

## Data Availability

The datasets generated and/or analyzed during the current study are available from the corresponding author on reasonable request.
