# Supplementary material for: Tumor Treating Fields Enhance Temozolomide‐Associated Growth Suppression in Glioblastoma Cells and Patient‐Derived Organoids Through Cell‐Cycle Blockade, Apoptosis, and SMAD‐Associated Signaling
Source: CNS Neurosci Ther. 2026 Oct 9;32(10):e71195. doi: 10.1002/cns.71195 (PMC13651720; doi:10.1002/cns.71195)
Supplement: Supplementary file 1 [file CNS-32-e71195-s001.zip › supplement.docx]

**Tumor Treating Fields Enhance Temozolomide-Associated Growth Suppression in Glioblastoma Cells and Patient-Derived Organoids Through Cell-Cycle Blockade, Apoptosis, and SMAD-Associated Signaling**

Haiping Zhu^1*^, Yingbo Hou^1*^, Xuetao Li^1^, Yi Tang^1^, Zuoyu Jiang^1^, Yang Zhang^1^, Xuewen Zhang^1^, Jiaming Du^1^, Yang Zhu^1^, Hanmiao Dong^1^, Yeyang Xu^1^, Ruoyu Sun^1^, Chenyang Li^1^, Zijian Han^1^, Jian Lu^2^, Lingzhi Xiong^2^, Yulun Huang^1†^

1. Department of Neurosurgery, The Fourth Affiliated Hospital of Soochow University, Suzhou, China.
2. Hunan An Tai Kang Cheng Biotechnology Co., Ltd., Changsha, Hunan, China

^*^Haiping Zhu and Yingbo Hou contributed equally.

^†^Correspondence to:

Dr. Yulun Huang: Email: huangyulun@suda.edu.cn

**Supplementary Figure S1. TTF parameter validation and wound-healing analysis in GBM cells.** (A) Frequency-response validation of TTF in U251 and SHG140 cells exposed to 100, 200, or 300 kHz at a fixed field intensity of 1.75 V/cm RMS for 48 h. Relative cell viability was assessed using the CCK-8 assay and normalized to the corresponding non-TTF control group. (B) Intensity-response validation of TTF in U251 and SHG140 cells exposed to 200 kHz TTF at 0.5–3.0 V/cm RMS for 48 h. Relative cell viability was assessed using the CCK-8 assay and normalized to the corresponding non-TTF control group. (C) Representative wound-healing images of U251 and SHG140 cells treated with vehicle control, TMZ, TTF, or TMZ+TTF at 0, 24, and 48 h. (D) Quantitative analysis of wound closure percentage measured using ImageJ. Because wound closure can be influenced by cell migration, proliferation, and treatment-related changes in cell survival, these results were interpreted as supportive evidence of migration-related changes. Five replicate wells were averaged within each parameter-validation experiment. For wound-healing analysis, three images per treatment group and time point were averaged within each independent experiment. Data are presented as mean ± SD (n = 3 independent experiments). Parameter-validation assays were analyzed using one-way ANOVA followed by Dunnett’s multiple comparisons test within each cell line. Wound-closure data were analyzed using two-way ANOVA followed by Sidak’s multiple comparisons test. *p < 0.05, **p < 0.01, ***p < 0.001, ****p < 0.0001.

**Supplementary Figure S2. TTF treatment alters the transcriptomic landscape of U251 glioblastoma cells.** (A) Boxplot showing the distribution of gene expression levels between vehicle control and TTF-treated U251 cells. (B) Principal component analysis (PCA) based on variance-stabilized counts from DESeq2, showing separation between vehicle control and TTF-treated samples and clustering of biological replicates within each group. (C) Volcano plot of differentially expressed genes between vehicle control and TTF-treated U251 cells. Upregulated genes are shown in red, downregulated genes in blue, and non-significant genes in grey. (D) GO enrichment analysis of differentially expressed genes categorized into biological process (BP), cellular component (CC), and molecular function (MF). (E) KEGG and Reactome enrichment analyses showing representative pathways associated with chromatin-related processes, transcriptional regulation, DNA replication, and cell-cycle regulation. Differentially expressed genes were defined using thresholds of |log₂FC| > 1 and false discovery rate (FDR) < 0.05. RNA-seq was performed using three independent biological replicates per group.

**Supplementary Figure S3. TTF treatment is associated with downregulation of DNA replication and cell-cycle pathways and G2/M-phase accumulation.** (A–C) GO enrichment analyses of downregulated genes in the biological process (BP), cellular component (CC), and molecular function (MF) categories. (D) Reactome enrichment analysis showing downregulated pathways related to DNA replication, DNA strand elongation, G1/S transition, and related biological processes. (E) KEGG pathway enrichment analysis showing downregulated pathways associated with DNA replication and cell-cycle regulation. (F) Representative flow cytometry histograms showing cell-cycle distribution in control and TTF-treated cells at 24 h and 48 h. (G) Quantitative analysis of cell-cycle phase distribution, showing increased G2/M-phase accumulation after TTF treatment. At least 10,000 events were acquired per sample in each of three independent experiments. Data are presented as mean ± SD (n = 3 independent experiments). Statistical analysis was performed using two-way ANOVA followed by Sidak’s multiple comparisons test. *p < 0.05, **p < 0.01, ***p < 0.001, ****p < 0.0001.

**Supplementary Figure S4. Western blot analysis of DNA damage checkpoint- and apoptosis-related markers in glioblastoma cells.** (A) Representative Western blots of phosphorylated CHK1 (p-CHK1), BIM, and PUMA in U251 and SHG140 cells treated with vehicle control, TMZ, TTF, or TMZ+TTF. GAPDH served as the loading control for p-CHK1, and β-tubulin served as the loading control for BIM and PUMA. (B, C) Quantitative analysis of protein expression levels in U251 and SHG140 cells, respectively. Data are presented as mean ± SD (n = 3 independent experiments). Statistical significance was determined using one-way ANOVA followed by Tukey’s multiple comparisons test. *p < 0.05, **p < 0.01, ***p < 0.001; ns, not significant.

**Supplementary Figure S5. Immunofluorescence analysis of apoptosis- and proliferation-related markers in GBM patient-derived organoids under different treatments.** (A, B) Representative immunofluorescence images of cleaved caspase-3 (red) and quantitative analysis of fluorescence intensity in vehicle control, TMZ, TTF, and TMZ+TTF groups. (C, D) Representative immunofluorescence images of Ki-67 (green) and quantitative analysis of fluorescence intensity across the same treatment groups. Nuclei were counterstained with DAPI (blue). Scale bars: 100 μm; the same scale applies to all fluorescence images within each panel. Data are presented as mean ± SD from three independent PDO lines derived from three patients (n = 3 biological replicates). For each PDO line and treatment group, three organoids were analyzed, and one fluorescence image was quantified per organoid. The three organoid-level measurements were averaged to generate one PDO-line-level value before statistical analysis. Individual organoids and images were considered technical subsamples rather than independent biological replicates. Statistical analysis was performed using repeated-measures one-way ANOVA followed by Tukey’s multiple comparisons test, with PDO line treated as the matched biological replicate. **p < 0.01, ***p < 0.001, ****p < 0.0001; ns, not significant.
